# Assessment of malaria real-time PCR methods and application with focus on low-level parasitaemia

**DOI:** 10.1371/journal.pone.0218982

**Published:** 2019-07-05

**Authors:** Christel Gill Haanshuus, Kristine Mørch, Bjørn Blomberg, Gro Elizabeth Ann Strøm, Nina Langeland, Kurt Hanevik, Stein Christian Mohn

**Affiliations:** 1 Norwegian National Advisory Unit on Tropical Infectious Diseases, Department of Medicine, Haukeland University Hospital, Bergen, Norway; 2 Department of Clinical Science, University of Bergen, Bergen, Norway; 3 Department of Medicine, Haukeland University Hospital, Bergen, Norway; 4 Haraldsplass Deaconess Hospital, Bergen, Norway; Universidade Federal de Minas Gerais, BRAZIL

## Abstract

In epidemiological surveys and surveillance the application of molecular tools is essential in detecting submicroscopic malaria. A genus-specific conventional cytochrome *b* (*cytb*) PCR has shown high sensitivity in field studies, detecting 70% submicroscopic malaria. The main objective of this study was to assess the conversion from conventional to real-time PCR testing both SYBR and probe protocols, and including quantitative (q) PCR. The protocols were assessed applying well-defined clinical patient material consisting of 33 positive and 80 negative samples. Sequencing of positive PCR products was performed. In addition, a sensitivity comparison of real-time PCR methods was done by including five relevant assays investigating the effect of amplification target and platform. Sensitivity was further examined using field material consisting of 111 *P*.*falciparum* positive samples from Tanzanian children (< 5 years), as well as using related patient data to assess the application of q-PCR with focus on low-level parasitaemia. Both the *cytb* SYBR and probe PCR protocols showed as high sensitivity and specificity as their conventional counterpart, except missing one *P*. *malariae* sample. The SYBR protocol was more sensitive and specific than using probe. Overall, choice of amplification target applied is relevant for achieving ultra-sensitivity, and using intercalating fluorescence dye rather than labelled hydrolysis probes is favourable. Application of q-PCR analysis in field projects is important for the awareness and understanding of low-level parasitaemia. For use in clinical diagnosis and epidemiological studies the highly sensitive and user-friendly *cytb* SYBR q-PCR method is a relevant tool. The genus-specific method has the advantage that species identification by sequencing can be performed as an alternative to species-specific PCR.

## Introduction

Malaria is a major threat to global health with half the world’s population being at risk of getting infected. In 2016 there were 216 million malaria cases causing almost half a million deaths, particularly among children in Sub-Saharan Africa and India [1]. The World Health Organisation has identified 21 countries that may be able to eliminate malaria by 2020 [1]. Healthy, asymptomatic humans carrying malaria parasites in the blood represent a reservoir for transmission [2], and are a key challenge to the elimination of malaria [3, 4]. Polymerase chain reaction (PCR) methods have gained a strong foothold in research and epidemiology during the last two decades. PCR has shown to be superior in sensitivity and specificity compared to microscopy and rapid diagnostic tests (RDT) [5–8]. However, there is still need to further improve the performance of malaria PCR techniques and to develop quantitative methods for field studies, to address the issue of asymptomatic carriage of malaria, and to increase our understanding of malaria epidemiology which may help improve control and elimination strategies [4, 6].

Visualizing conventional PCR products by gel electrophoresis is time-consuming and resource-demanding, and increases the risk of contamination. With real-time PCR the detection step is incorporated into the amplification; for each cycle, target DNA is directly detected, usually by either intercalating SYBR green, or fluorescence labelled hydrolysis probes [9, 10]. The direct detection allows for quantitative results (q-PCR), presented either by standard cycle threshold (C_t_) values, which is inversely proportional to the amount of target DNA in the sample, or even more exact quantitation by applying a known dilution series of target DNA (for example customized plasmid) which serves as a standard curve to determine the number of target copies per volume in the sample. Thus, q-PCR could be a highly valuable tool to study low-level malaria parasitaemia [10, 11].

During the last decade, there has been a focus on the use of amplification targets with a high copy number in the malaria genome to increase the sensitivity of the PCR. Examples of these multi-targets are chromosomal small subunit ribosomal RNA 18S locus, cytochrome b gene (*cytb*) on the mitochondrial genome, the chromosomal subtelomeric targets telomere-associated repetitive element 2 (TARE-2) and *var* gene acidic terminal sequence (*var*ATS). The first malaria PCR methods were based on the 18S target, which typically exists in five to eight copies in *P*. *falciparum* [12]. Subsequently, mitochondrial targets, such as *cytb*, emerged since the genome is conserved and exists in about 20–160 copies depending on the development stage [13, 14]. In 2015 two new multi-targets were reported; TARE-2, which exists in about 250–280 copies due to 10 to 12 repeat units presented at 24 of 28 subtelomeric sequences, and *var*ATS which exists in about 59 copies and is encoding the *P*. *falciparum* erythrocyte membrane protein 1 [15].

A genus-specific *cytb* conventional PCR [16] has proved to be highly sensitive and specific in field studies [5, 17]. The main objective of the present study was to develop a robust screening tool by converting the conventional PCR to a real-time PCR, evaluate two different fluorescent dyes SYBR green and TaqMan probe, and to assess the assays using well-defined patient materials from clinical and field collections [5, 16, 18]. Secondary objectives were to compare relevant real-time methods in order to investigate the effect the choice of platform (SYBR versus probe) and amplification target has on sensitivity, and to implement q-PCR analysis on patient data from field material with focus on low-level parasitaemia.

## Materials and methods

### Samples

All sample material applied in this study was stored DNA (-20/80°C), which had been extracted either from whole blood using QIAamp DNA Blood Mini Kit (Qiagen, Hilden, Germany) according to the manufacturer’s instructions, or from filter paper applying Chelex-100 Molecular Biology Grade Resin (Bio-Rad Laboratories, Hercules, CA, USA) as previously described [19].

The clinical patient material, used to assess the sensitivity and specificity of the designed *cytb* SYBR/TaqMan real-time assays, was a defined collection of 33 confirmed positive (18 *P*. *falciparum*, eight *P*. *vivax*, three *P*. *ovale*, two *P*. *malariae*, and two *P*. *falciparum* + *P*. *malariae*), and 80 confirmed negative samples, from 113 fever patients with potential malaria collected between 2006 and 2013 at Haukeland University Hospital, Bergen, Norway [16]. The consensus on which samples were positive or negative was based on results from previously performed analyses by routine microscopy, the PCR reference method described by Singh *et al*. [20], a genus-specific *cytb* PCR, a species-specific 18S PCR (all conventional), and sequencing [16].

The field material, used to investigate sensitivity and to perform q-PCR, was a defined collection of 111 positive *P*.*falciparum* DNA samples (74 patients), where 74 were extracted from 200 μl EDTA whole blood, and 37 from filter papers. The DNA was obtained from 304 children who were hospitalized due to febrile illness between January and June 2009 at the general pediatric wards at Muhimbili National Hospital, Dar es Salaam, Tanzania [5, 18]. The samples were confirmed positive by the same genus-specific *cytb*, and species-specific 18S conventional PCR assays (or sequencing) as the Norwegian cohort [16]. Clinical and demographic data, such as age, gender, travel outside Dar es Salaam the last four weeks, referral from other hospitals, any use of antibiotics or antimalarial drugs the last four weeks, length of sickness, other clinical diagnoses, and outcome, were included in the analysis of potential correlation with level of parasitaemia by q-PCR. Details regarding the study population and clinical findings in the Tanzanian field study have been reported previously [5].

### Reference material

To compare the sensitivity of seven different real-time PCR methods a reference strain of *P*. *falciparum* (US 03 F FC27/A3), containing exclusively ring stage parasites in a concentration of 2000 parasite/μl (p/μl), was diluted in RNase-free water (Qiagen) into the following dilutions: 5 p/μl, 1 p/μl, 0.5 p/μl, 0.1 p/μl, and 0.05 p/μl, run in 12 parallels. In addition, a 10-fold dilution series run in triplicates, 2000–0.2 p/μl, was included to assess the amplification efficiency (E) of the real-time assays.

### Customized plasmid for q-PCR

To serve as a standard curve for q-PCR, a custom designed EcoRI linearized q-PCR template, with a pUCminusMCS vector backbone and this study’s *cytb* amplification target (220 bp) as insert (OriGene Technologies, Rockville, MD, USA), was applied in a 10-fold dilution series, range 2.7 x 10^8^–2.7 copies of target DNA/reaction (rxn), run in duplicates.

### PCR methods

A conventional single-step genus-specific *cytb* PCR [16] was converted to one SYBR and one TaqMan real-time PCR protocol using the same primers.

To investigate sensitivity differences/trends, and effects the number of copies of the amplification targets may have, five relevant and comparable real-time PCR methods were included. Table 1 shows the characteristics of the real-time PCR assays.

**Table 1:** Characteristics of real-time PCR methods.

Methods	Specificity	Target genome	Target gene	No. copy of gene in P.f	Product size	Species identification by sequencing
**This study**	Pan	Mito	*cytb*	~20–160^a^	220 bp	Yes
**Lefterova** et al. **2015** [21]	Pan	Chrom	18S rRNA	~5–8^b^	317 bp	Yes
**Xu** et al. **2015** [22]	Pan	Mito	*cytb*	~20–160^a^	430 bp	No^e^
**Farrugia** et al. **2011 [23]**	Pan	Mito	*cytb*	~20–160^a^	203 bp	No
**Hofmann** et al. **2015** [15]	P.f	Chrom	TARE-2	~250–280^c^	93 bp	-
**Hofmann** et al. **2015 [15]**	P.f	Chrom	*var*ATS	~59^d^	65 bp	-

**Abbreviations:** P.f, *Plasmodium falciparum*; Mito, mitochondrial; Chrom, chromosomal; *cytb*, cytochrome *b* gene; telomere-associated repetitive element 2, TARE-2; *var* gene acidic terminal sequence, *var*ATS.

^a^ Depending on which stage of the parasite cycle; About 20 copies in early ring stage, and about 80–120 copies in mature gametocytes [13, 14].

^b^ Depending on the strain [12].

^c^ TARE-2, specific to *P*. *falciparum*, consists of 10 to 12 repeat units presented at 24 of 28 subtelomeric sequences [15].

^d^ The *var* gene family is located primarily in the subtelomeric sequences, and encode the *P*. *falciparum* erythrocyte membrane protein 1 [15].

^e^ Species identification can be performed using restriction fragment length polymorphism analysis of the real-time PCR amplified product [22].

To optimize their comparability, all the real-time assays applied 2 μl DNA template, and 12,5 μl SYBR Select Master Mix/ TaqMan Universal Master Mix II, with UNG (Applied Biosystems, Carlsbad, CA, USA), at a total volume of 25 μl. The amplifications were performed using ABI Prism 7900HT Sequence Detection System (Applied Biosystems), the threshold was automatically set, and for the SYBR assays melting curve analysis was included given by the program SDS 2.3 (Applied Biosystems).

Otherwise, the protocols for the five included real-time PCR methods were carefully followed as previously described [15, 21–23].

For the *cytb* SYBR/TaqMan real-time PCR protocols designed and optimized in this study the following primers were applied: PgMt19 F3 forward (5’-tcg ctt cta acg gtg aac) and PgMt19 B3 reverse (5’-aat tga tag tat cag cta tcc ata g), previously published for a loop-mediated isothermal amplification method [24]. The primer concentration was 600 nM of each primer, and 200 nM of the TaqMan probe PgMt(28)-Probe 6-FAM-ctt cta aca ttc cac ttg ctt ata act g-BHQ-1 (Eurogentec, Seraing, Belgium). In addition, the reaction mix contained 1 mM MgCl_2_ (New England BioLabs, Ipswich, MA, USA). Both protocols used the following cycling parameters; step 1, 50°C for 2 min; step 2, 95°C for 10 min; step 3, denaturation at 95°C for 15 sec; step 4, annealing at 59°C for 50 sec; and step 5, amplification at 72°C for 10 sec, steps 3–5 repeated 45 times.

To investigate the quality of the stored DNA, samples of extremely low parasitaemia were reanalysed with the *cytb* conventional PCR (primers PgMt19 F3&B3), run in triplicates, as previously described by Haanshuus *et al*. [16], but with a primer concentration of 1 μM (incorrect concentration given in the publication [16]).

To ensure that real-time PCR products showed the same high quality sequences for species identification as for the *cytb* conventional PCR [16], all positive products amplified by the *cytb* SYBR real-time PCR from the Norwegian material, were sequenced in one direction applying primer PgMt19 F3 as previously described [16].

### Statistical analyses

Statistical univariate analysis was performed applying IBM SPSS Statistics version 24 (SPSS Inc., IBM Company). The data were organized into categorical variables for 2x2 cross-tabulation analysis, and assessed with effect estimates (odds ratio) with corresponding confidence intervals, as well as Chi-squared test, or Fisher’s exact test if few observations. A correlation was regarded as statistically significant if p-value was < 0.05.

Multivariate logistic regression analysis was also performed applying SPSS. The p-values were calculated by using the Likelihood ratio test, where in addition to SPSS the program QuickCalcs (GraphPad Software) was applied for this purpose. All variables with p-value <0.1 from the univariate analysis, excluding variables with extensive numbers of missing values, were included in the multivariate regression model. The confirmation that the model fitted the data was evaluated by the Hosmer and Lemeshow Test, and by residuals and Cook distances analysis.

### Ethical approval

The study was approved by the Regional Committee for Ethics in Medical Research in Western Norway (No.2015/886 and 2016/584).

## Results

All raw data, including threshold, C_t_- and quantification values, are given in supporting information S1 Dataset. The clinical and field DNA samples were always run in triplicates, and a positive result was defined as minimum two detections out of three.

### Assessment, comparability, and sensitivity of real-time PCR methods

The sensitivity and specificity of the designed and optimized genus-specific *cytb* SYBR and TaqMan PCR protocols were assessed applying the Norwegian clinical material. The real-time assays showed as high sensitivity and specificity as their conventional PCR counterpart [16], except missing one *P*. *malariae* sample (S1 Table).

Applying *P*. *falciparum* reference material, the study’s two protocols and five relevant real-time PCR methods (characteristics presented in Table 1) were compared to investigate how the choice of platform and amplification target affects the sensitivity (Tables 2 and S2).

**Table 2:** Sensitivity comparison of real-time PCR methods applying five different dilutions of *P*.*falciparum* reference material. ^**a**^.

	Platform	5 p/μl (${\overset{¯}{C}}_{t}$)	1 p/μl (${\overset{¯}{C}}_{t}$)	0.5 p/μl (${\overset{¯}{C}}_{t}$)	0.1 p/μl (${\overset{¯}{C}}_{t}$)	0.05 p/μl (${\overset{¯}{C}}_{t}$)
**This study**_*cytb*	SYBR	12 (30)	12 (33)	12 (34)	9 (36)	9 (38)
**This study**_*cytb*	TaqMan	12 (30)	12 (32)	12 (33)	8 (35)	5 (37)
**Lefterova**_18S	SYBR	12 (28)	12 (32)	11 (34)	4 (36)	0
**Xu**_*cytb*	SYBR	12 (28)	12 (32)	12 (33)	10 (35)	6 (35)
**Farrugia**_*cytb*	TaqMan	12 (34)	12 (37)	12 (38)	3 (41)	1 (42)
**Hofmann**_TARE-2	SYBR	12 (33)	12 (37)	12 (38)	7 (40)	3 (39)
**Hofmann**_*var*ATS	TaqMan	12 (34)	12 (36)	12 (37)	11 (40)	8 (41)

^a^ The threshold was automatically set for each assay run, and the C_t_ values correspond to the threshold. The results are given as the number of positives out of 12 parallels.

**Fig 1:**
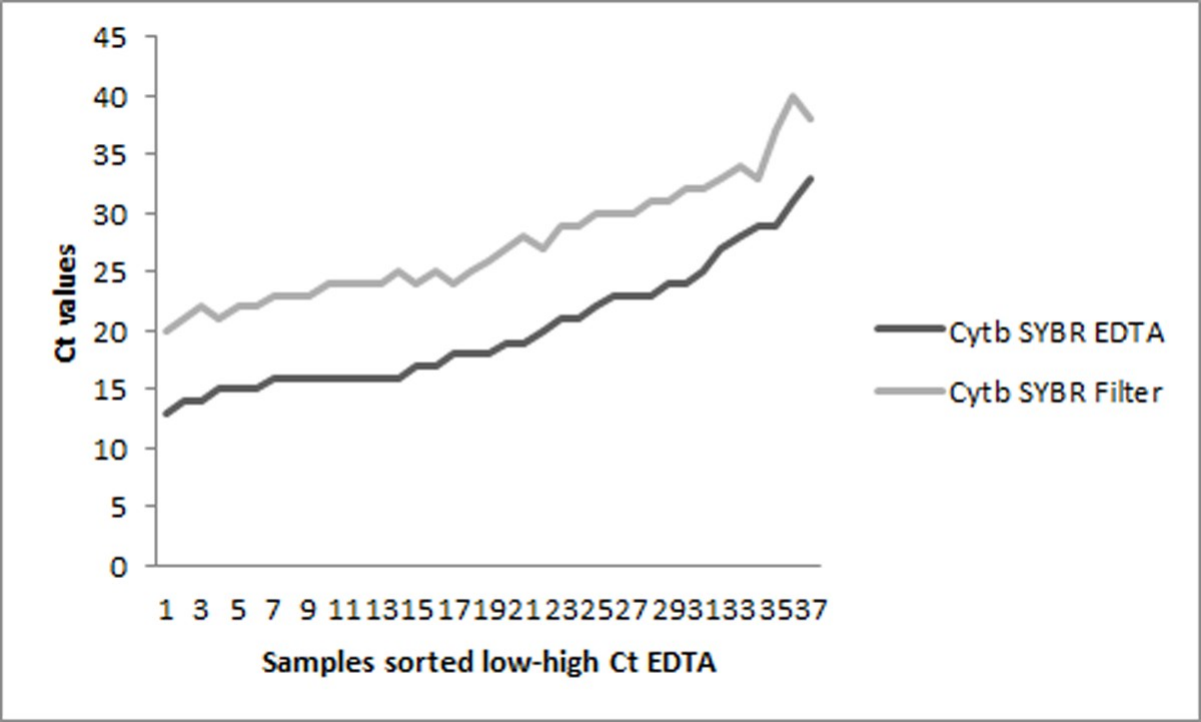
C_t_ value comparisons of DNA samples extracted from 200 μl EDTA whole blood versus filter paper 50 μl blood (N = 37). Positive *P*.*falciparum* material from 37 patients had been collected and extracted by two different methods resulting in DNA from whole blood purified by spin-column, and DNA from filter paper purified by Chelex-100 [5, 18]. The two C_t_ trends showed a constant difference of 4–9 cycles, and for 31 out of 37 samples the difference was 7–9 cycles.

**Fig 2:**
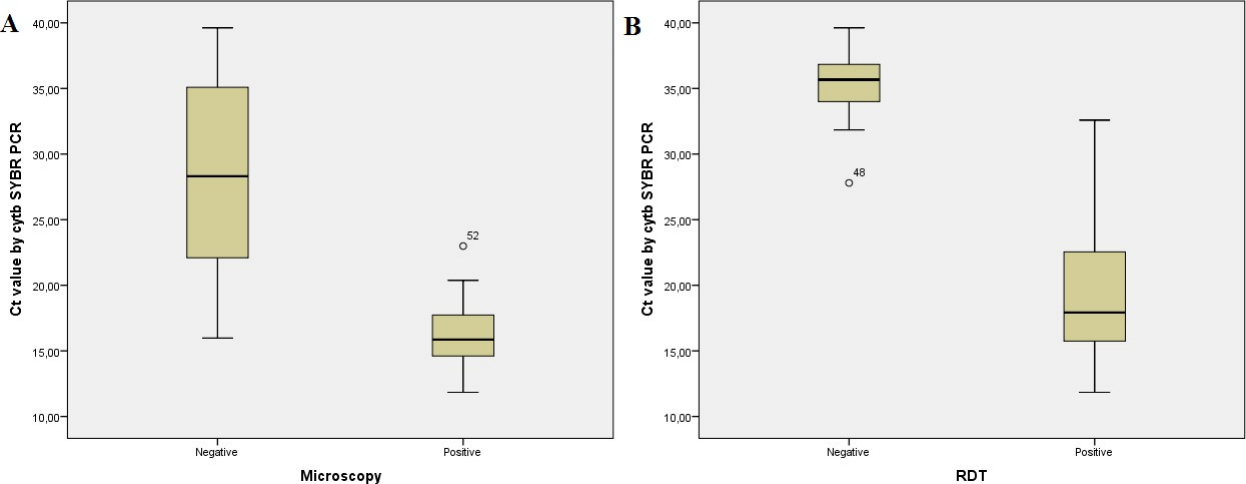
Boxplots showing how research microscopy and RDT results correlate to positive C_t_ values among Tanzanian field samples. (A) A boxplot showing how research microscopy results negative/positive correlate to positive *cytb* SYBR real-time PCR results (N = 54). (B) A boxplot showing how rapid diagnostic test (RDT) results negative/positive correlates to positive *cytb* SYBR real-time PCR results (N = 47).

Among the five included assays the *var*ATS TaqMan PCR method [15] showed a high sensitivity, and was therefore chosen for further assessment together with the designed *cytb* SYBR/TaqMan PCR protocols, applying *P*.*falciparum* positive Tanzanian field material (Table 3). Furthermore, the sensitivity of applying whole blood versus that of using filter paper as field material was compared, and the difference in C_t_ trends for the *cytb* SYBR real-time PCR results are shown in Fig 1. Among the field samples extracted from whole blood, previously obtained research microscopy and RDT results were correlated with positive C_t_ values of the *cytb* SYBR PCR as presented in Fig 2.

**Table 3:** Sensitivity assessment of real-time PCR methods applying positive *P*. *falciparum* Tanzanian field DNA samples (N = 111). ^**a**^.

		This study Pan_*cytb*_SYBR	This study Pan_*cytb*_TaqMan	Hofmann et al. *P*.*f*_*var*ATS_TaqMan
**Samples** **EDTA**^**b**^ **(N = 74)**	**Positives C**_**t**_ **< 20**	25	20	19
	**Positives C**_**t**_ **20–30**	16	19	18
	**Positives C**_**t**_ **31–40**	13	15	24
	**Positives C**_**t**_ **> 40**	0	0	0
	**Positives total**	54	54	61
	**Negatives**	20	20	13
**Samples** **Filter**^**c**^ **(N = 37)**	**Positives C**_**t**_ **< 20**	0	0	0
	**Positives C**_**t**_ **20–30**	27	23	26
	**Positives C**_**t**_ **31–40**	10	11	8
	**Positives C**_**t**_ **> 40**	0	0	1
	**Positives total**	37	34	35
	**Negatives**	0	3	2

**Abbreviations:** P.f, *Plasmodium falciparum*

^a^ Predefined samples by genus-specific *cytb* conventional PCR, and species-specific 18S PCR (or sequencing) [5, 18]. Data on concluding positivity is given in S1 Dataset.

^b^ DNA extracted from 200 μl EDTA whole blood by a spin-column method.

^c^ DNA extracted from filter paper (~50 μl blood) by a Chelex-100 method.

**Fig 3:**
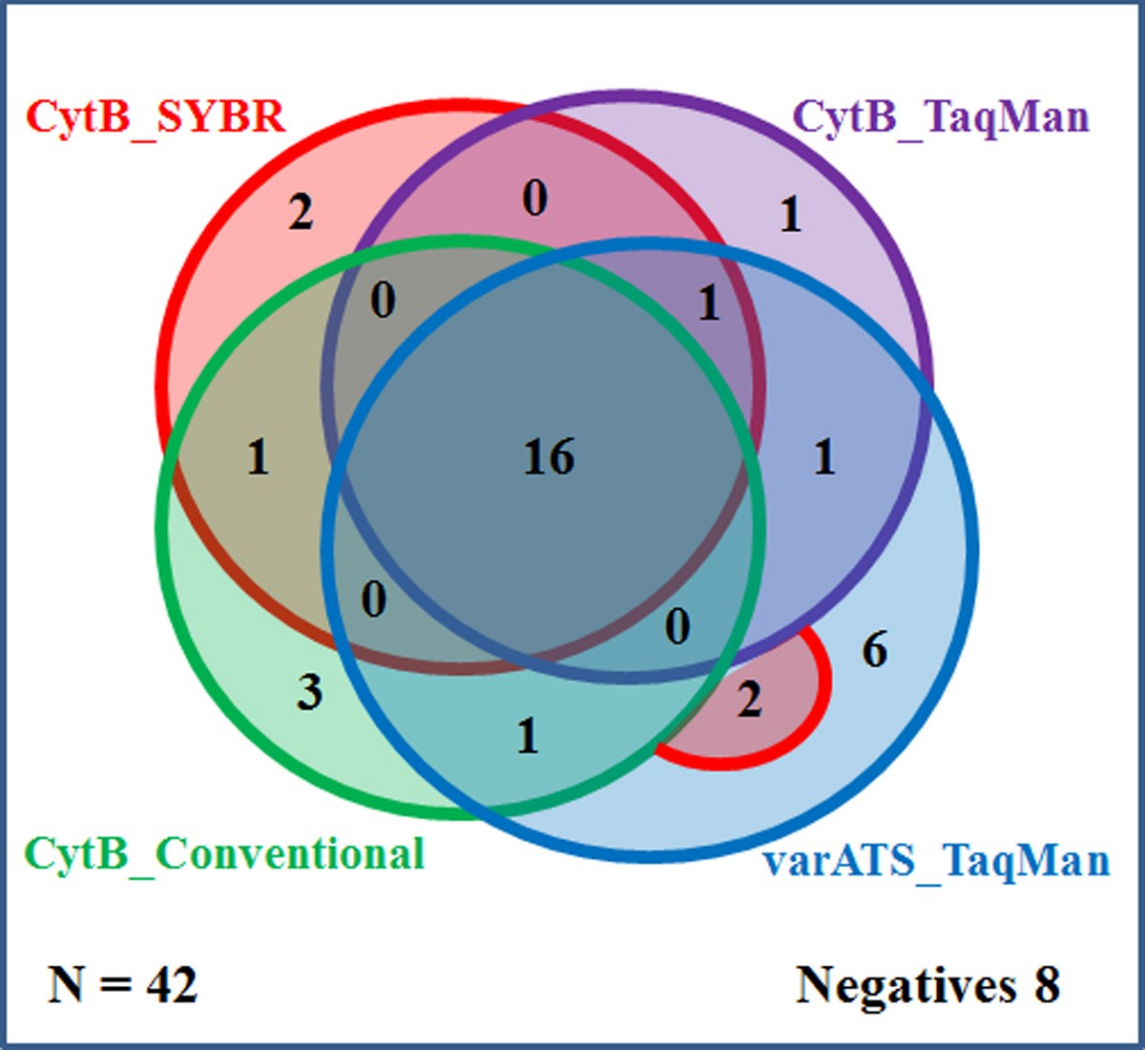
Sensitivity analysis and method comparison of PCR methods applying positive *P*.*falciparum* field DNA samples with extreme low parasitaemia (N = 42). Extreme low parasitaemia samples that counted negative or C_t_ value above 30 by *cytb* SYBR real-time PCR were further analyzed. This was done to investigate if the stored DNA had been degraded over time, as well as compare the performance of the four PCR methods. The *var*ATS TaqMan real-time PCR had a higher detection rate for low parasitaemia. However, for all of the methods eight of the previously positive *P*. *falciparum* samples were now negative. A positive sample was defined by at least two out of three parallels detected, and only one of the eight negatives had no positive detections of parallels by any of the methods. For several of the samples it was a randomly/non-consistently detection trend, depending on the actual amount of amplification target in the template. Detailed data are given in S1 Dataset.

Through the analysis of both the Norwegian and Tanzanian material, several of the previously positive DNA samples turned out negative by the real-time PCR methods. Therefore, the quality of the stored DNA was tested by reanalysing, with the *cytb* conventional PCR [5, 16, 18], all samples which were negative or had a C_t_ value above 30 by the *cytb* SYBR real-time method. Fig 3 shows a comparison between the conventional and the real-time PCR methods.

The designed *cytb* SYBR PCR assay showed better results than using TaqMan probe (Tables 2 and 3 and S1 and S2). Therefore this method was chosen for the q-PCR analysis of the Tanzanian field material, and to test the quality of sequencing as species identification method. The sequencing confirmed high-quality sequences of full length, and gave correct species identification, except for only detecting the dominating *P*. *falciparum* parasitaemia in two double infections. This result was equal to that of applying the conventional PCR version, and the polymorphisms distinguishing the species were previously described [16].

### Application and assessment of quantitative PCR field data

To present an alternative and relevant application of q-PCR data in epidemiological studies, the association between low-level parasitaemia identified by the *cytb* SYBR q-PCR results and clinical and demographic factors (among the 74 field samples extracted from whole blood), were evaluated applying cross tabulation and multiple logistic regression analysis. Based on an evaluation of C_t_ results, frequency data, and what was of low density parasitaemia (S1 and S2 Datasets), the q-PCR values were categorized into the two categories ≤1000 copies/rxn (N = 37, range 0–953, corresponds to C_t_ value ≥27) defined as low parasitaemia, and > 1000 copies/rxn (N = 37, range 3769–1.6x10^7^, corresponds to C_t_ value ≤24) defined as high parasitaemia. The median value was 2361 copies/rxn. When applying 2 μl template, 1000 copies of the *cytb* target/rxn, corresponds to 3–25 *P*. *falciparum* p/μl depending on the parasite stage (Table 1). Microscopy had the most sensitive detection at C_t_ value 23 and 15314 copies/rxn, which corresponds to ~50–380 p/μl. In comparison, according to Hänscheid et al. 1% parasitaemia is measured to be ~50.000 p/μl [25].

In the univariate analysis (Table 4), low-level parasitaemia was associated with age ≤12 months, illness of >5 days duration, and death while in hospital. Patients with low-level parasitaemia were more likely to have received antibiotics the last four weeks, but less likely to have been diagnosed with malaria, and less likely to have received antimalarial treatment in hospital. In the multivariate analysis (S3 Table), low level parasitaemia was associated with age ≤12 months, length of illness >5 days, no history of travel outside Dar es Salaam the last four weeks, and not being diagnosed with malaria. The variables antibiotic and antimalarial treatment in hospital were omitted from the regression model due to a high number of missing values.

**Table 4:** Univariate cross tabulation analysis of factors associated with low parasitaemia (≤1000 copies/rxn).

	Low parasitaemia ≤ 1000 copies/rxn (%)	High parasitaemia > 1000 copies/rxn (%)	OR (95% CI)	p-value
*Age (N = 73)*				
≤ 12 months	17 (47.2)	5 (13.5)	5.73 (1.82–18.04)	0.002*
> 12 months	19 (52.8)	32 (86.5)		
*Sex (N = 74)*				
Male	22 (59.5)	19 (51.4)	1.39 (0.55–3.49)	0.483
Female	15 (40.5)	18 (48.6)		
*Travel outside Dar last 4 weeks (N = 63)*				
No	19 (65.5)	15 (44.1)	2.41 (0.87–6.69)	0.089
Yes	10 (34.5)	19 (55.9)		
*Referral from other hospital (N = 71)*				
No	24 (68.6)	29 (80.6)	0.53 (0.18–1.57)	0.246
Yes	11 (19.4)	7 (19.4)		
*Antibiotics the last 4 weeks (N = 66)*				
No	5 (14.3)	15 (48.4)	0.18 (0.06–0.58)	0.003*
Yes	30 (85.7)	16 (51.6)		
*Antimalarials the last 4 weeks (N = 68)*				
No	15 (44.1)	11 (32.4)	1.65 (0.62–4.43)	0.318
Yes	19 (55.9)	23 (67.6)		
*Length of sickness (N = 72)*				
≤ 5 days	16 (43.2)	29 (82.9)	0.16 (0.05–0.47)	0.001*
> 5 days	21 (56.8)	6 (17.1)		
*Antibiotic treatment in hospital (N = 74)*				
No	2 (5.4)	4 (10.8)	0.47 (0.08–2.75)	0.219
Yes	35 (94.6)	33 (89.2)		
*Antimalarial treatment in hospital (N = 74)*				
No	8 (21.6)	1 (2.7)	9.93 (1.17–84.04)	0.008*
Yes	29 (78.4)	36 (97.3)		
*Given diagnosis malaria (N = 74)*				
No	22 (59.5)	10 (27.0)	3.96 (1.49–10.53)	0.005*
Yes	15 (40.5)	27 (73.0)		
*Given diagnosis septicaemia (N = 74)*				
No	24 (67.6)	32 (86.5)	0.33 (0.10–1.05)	0.053
Yes	12 (32.4)	5 (13.5)		
*Length of admission (N = 74)*				
≤ 5 days	24 (64.9)	20 (54.1)	1.57 (0.62–4.00)	0.343
> 5 days	13 (35.1)	17 (45.9)		
*Outcome (N = 74)*				
Dead	11 (29.7)	4 (10.8)	3.49 (1.00–12.24)	0.043*
Alive	26 (70.3)	33 (89.2)		

Abbreviation: OR Odds ratio, 95%CI 95% Confidence Interval, *Dar* Dar es Salaam.

*Significant results (p-value < 0.005).

## Discussion

Several studies report that PCR techniques are superior to microscopy, especially in detecting low density parasitaemia [26–28]. A review of studies comparing PCR and microscopy, found that PCR detects on average twice as many malaria infections as microscopy [29]. The genus-specific *cytb* conventional PCR [16], has previously been applied in studies from Tanzania and India, where the PCR detected as much as 72% (55/76, N = 304) and 71% (162/228, N = 1168) submicroscopic malaria respectively [5, 17]. The conversion and optimization from conventional to real-time PCR, using either SYBR green or TaqMan probe, resulted in similar sensitivity and specificity (S1 Table and Fig 3 and S1 Dataset).

Applying SYBR is less expensive than using probes. Though the specificity of SYBR assays is debated; while probes bind specifically to target DNA as an third oligo, the specificity of SYBR relies on a high efficiency and concentration of specific amplified DNA outshining the unspecific binding of SYBR [30]. Short amplicons give higher efficiencies. In contrast to probes, which release only a single fluorophore for each amplicon, SYBR will with longer amplicons generate a stronger signal increasing the sensitivity as more dye is incorporated [31]. This is in agreement with the observed higher sensitivity of the study’s SYBR assay compared to using probe (Tables 2 and 3 and S1 and S1 Datasets). Using SYBR showed 100% specificity, while the TaqMan assay had one unspecific binding at C_t_ value 41 (S1 Table). The SYBR protocol in this study is recommended compared to using TaqMan probe.

Comparing different real-time PCR methods showed that the assays are remarkably similar in sensitivity (Table 2). The 18S SYBR PCR (Lefterova *et al*. [21]) had the lowest sensitivity, as might be expected since the target gene is in only a few copies (Table 1). Surprisingly, the method using the target in most copies, TARE-2 SYBR PCR (Hofmann *et al*. [15]), was not the most sensitive one. However, this agrees with the authors’ report of lower sensitivity than expected, likely due to the degenerate sequence of the TARE-2 repeat units or by the clustered distribution of the repeats at chromosome ends. The three most sensitive assays were the *var*ATS TaqMan (Hofmann *et al*. [15]), the *cytb* SYBR (Xu *et al*. [22]), and this study’s *cytb* SYBR PCR. The *cytb* SYBR assay from Xu *et al*. showed the lowest amplification efficiency (S1 Table), which is in agreement with the authors’ report on a secondary structure of the main amplicon that likely contributes to a competing binding-site for the primers, and can affect optimal sensitivity. This study’s assays, the 18S SYBR (Lefterova *et al*.), and the *cytb* SYBR (Xu *et al*.), are genus-specific PCR methods which have the advantage of species identification by either sequencing or restriction fragment length polymorphism analysis. The TARE-2 and *var*ATS methods are restricted to *P*.*falciparum*. In clinical use any of these PCR assays will be sensitive enough; however, in detecting low-level parasitaemia the small differences in sensitivity might be relevant. Furthermore, amplification in late cycles of PCR programs can be due to unspecific binding or primer-dimers. However, it was in this study decided to not set a cut-off for positivity as also low-level parasitaemia has late amplifications (Table 2). Hence the SYBR methods were more beneficial to apply than TaqMan probes focusing in detecting low-level parasitaemia, since SYBR melting curve analysis (MCA) can be performed revealing if late cycle amplifications are unspecific binding or primer-dimers rather than true positives [32].

PCR detects very low densities of target DNA in the blood (S1 and S2 Datasets), and compared to microscopy and RDT, the technique has a superior sensitivity in detecting low-level parasitaemia (Fig 2). Asymptomatic malaria among PCR positives should always be considered [17, 33]. To achieve a low detection limit, the type of material is important. Filter papers are increasingly used in field studies, as the material is easier to collect, store, and transport than EDTA whole blood, but result in lower test sensitivity due to the smaller blood volume obtained, ~50 versus 200 μl (Fig 1) [18]. Target DNA extracted from filter papers had in this study a detection limit of 25 copies/rxn, while DNA from EDTA had 1 copy/rxn. Therefore, applying filter papers gives a high sensitivity, but a substantial portion of the low-level parasitaemia will be missed which might be relevant in surveillance and epidemiological studies.

The *P*. *falciparum* positive field material extracted from whole blood (N = 74), showed a broad variation in level of parasitaemia (Table 3). There is a great complexity of reasons why malaria DNA detected only by PCR is obtained in the blood stream. Low density of malaria DNA may derive from early (primary or recurrent) infection, non-*falciparum* infection (as indicated in S1 Table where a clinical *P*. *malariae* infection showed a high C_t_ value), premunition, or remains of parasites and denatured DNA after infection clearance by the immune system and antimalarial treatment. Also some antibiotics may reduce the parasite level in the blood without fully clearing the infection [34]. Sexual stage parasites (gametocytes) are less affected by antimalarial drugs and the immune system, and thus circulate in the blood for weeks after parasite life cycles are ended awaiting transmission [35, 36]. Untreated chronic low-level parasitaemia might persist for months as parasite clones can circulate for ~200 days [37].

Due to potentially reduced quality of the stored DNA, and low reproducibility for samples with extremely low parasitaemia (Fig 3), the field material (N = 111) was also used for sensitivity assessment of the the designed *cytb* SYBR/TaqMan and the *var*ATS TaqMan PCR methods. Compared to DNA extracted from whole blood, the DNA from filter paper showed high stability with only a few previously positive samples testing negative (Table 3), which may be caused by the ion‐exchange resin Chelex solution inhibiting DNA degradation by chelating metal ions that otherwise can catalyze breakdown of DNA [38]. Table 3 also showed that the *var*ATS assay detected five more positive samples than the *cytb* SYBR method. With regards to the two different amplification targets *cytb* versus *var*ATS, *cytb* PCR assays should in theory be twice as sensitive as *var*ATS PCR in detecting gametocytes, while *var*ATS targets should be about three times as sensitive as *cytb* in detecting ring stage parasites (Table 1). Therefore, it can be speculated if a substantial proportion the field samples with low-level parasitaemia are malaria infections dominated by asexual blood stage parasites, rather than gametocytes.

By applying a customized plasmid, quantitation analysis was performed, and the association between degree of parasitaemia defined by q-PCR values (N = 74) and clinical and demographic features was assessed (Tables 3 and S3). The study population was children <5 years. Children have a less developed immune system than adults, though antibodies from their mother and in breastmilk give some protection in their first months of life [39, 40]. Both the univariate and multivariate analysis found a significant correlation between age ≤12 months and low parasitaemia, which could be due to immunity and asymptomatic malaria in this group. Among children age ≤12 months, 37% (7/19) had received antimalarials within four weeks of admission, versus 71% (34/48) among children age >12 months (S2 Dataset). Furthermore, in the univariate analysis, low parasitaemia was significantly associated with death in hospital (Table 3), though not significant in the multivariate analysis (S3 Table). The association is counter-intuitive, as high parasitaemia is known to be a risk factor for death. However, low parasitaemia malaria may be wrongly interpreted as clinical malaria obscuring the diagnosis of other severe blood stream infections. In a previous study from the same hospital, bloodstream-infections caused by multi-resistant Gram-negative bacteria carried case-fatality rates (>70%) more than three-fold that of malaria (≈20%) [41]. The finding that patients with low-level parasitaemia less frequently had been given a diagnosis of malaria, and less frequently had received antimalarial treatment, support that other infections may have been the cause of disease and mortality in these children, or might indicate missed opportunity for treatment of early malaria infection. The DNA detected by PCR can stem from a recent malaria episode for which the patient had already received treatment and recovered from. Indeed, children who have recently been hospitalized with malaria and anemia have substantially increased risk of death in the subsequent months [42, 43]. Among the children 81% (51/63) had received either antibiotics 70% (46/66), antimalarials 62% (42/68), or both 46% (29/63), within four weeks prior to admission (S2 Dataset). A limitation to the q-PCR assessment is the small sample size and the limit age-interval (children <5 years), as well as few clinical parameters. The samples used in the assessment had been collected in order to perform malaria PCR, but for a different purpose and application (Strøm *et al*. investigating challenges in diagnosing paediatric malaria [5]). Therefore, to assess predictors of clinical outcome in low-parasitaemia, this alternative q-PCR approach should be applied in larger, prospective studies employing comprehensive diagnostics for the major relevant differential diagnoses of acute undifferentiated febrile illness, including blood culture and serology for rickettsia and viral infections. Furthermore, since an unknown portion of the low parasitaemias probably is due to gametocytes, methods detecting mRNA specifically produced by gametocytes could be relevant to include increasing knowledge around transmission dynamics [44].

Stored DNA is commonly used in PCR assessments and analysis, since extracted DNA is regarded as a fairly stable material if kept in a freezer [45]. It has been debated if freezing and thawing of samples can have a denaturing effect on the DNA [46]. The quality of the stored DNA in this study was tested by reanalyzing the samples with the *cytb* conventional PCR used in the first analyses of the Tanzanian field material [5]. The results indicated that the DNA might have been degraded over time (Fig 3 and S1 Dataset), which, is a possible bias to the correlations shown in Fig 2 and Table 4. However, Fig 1 demonstrates the accuracy and consistency of PCR, and shows no indication of denatured DNA in samples with high parasitaemia. For samples with extremely low parasitaemia, reproducibility by PCR is challenging (Fig 3 and S1 Dataset). The number of times each sample was defrosted varied due to testing, therefore some samples might have been more prone to degradation than others (S1 Dataset). Since all the 74 samples assessed with q-PCR were previously confirmed positive by PCR (data on concluding positivity is given in S1 Dataset), it was decided in regards to the patient data that also q-PCR results with value zero should be included in the definition of low parasitaemia (≤1000 copies/rxn).

### Conclusions

Choice of amplification target applied in real-time malaria PCR is relevant for achieving high sensitivity, especially in detecting low-level parasitaemia. Furthermore, it is advantageous to use an intercalating fluorescence dye suitable for MCA rather than labelled hydrolysis probes. Application of q-PCR analysis should be included in epidemiological studies, and may increase awareness and understanding of low-level parasitaemia. The highly sensitive, specific, and user-friendly *cytb* SYBR q-PCR developed in this study can be useful in both epidemiological and clinical malaria studies. The method is genus-specific, which is an advantage in large screening projects. Among ambiguous samples, and in settings where species-specific PCR fails to detect low parasitaemia, confirmation and species identification by sequencing of the genus-specific real-time PCR products can be used as a contingency.

## Supporting information

S1 Dataset Raw PCR data. (XLSX)

S2 Dataset Patient and q-PCR data. (SAV)

S1 Table Sensitivity and specificity assessment of designed real-time PCR protocols applying Norwegian clinical DNA samples (N = 113). (DOCX)

S2 Table Amplification efficiencies for real-time PCR methods applying a 10-fold dilution series of reference material. (DOCX)

S3 Table Multivariate logistic regression analysis of factors associated with low parasitaemia (≤1000 copies/rxn). (DOCX)
