# Supplementary material for: Assessment of malaria real-time PCR methods and application with focus on low-level parasitaemia
Source: PLoS One. 2019 Jul 5;14(7):e0218982. doi: 10.1371/journal.pone.0218982 (PMC6611585; doi:10.1371/journal.pone.0218982)
Supplement: S1 Table — (DOCX) [file pone.0218982.s003.docx]

**S1 Table Sensitivity and specificity assessment of designed real-time PCR protocols applying Norwegian clinical DNA samples (N=113)^a^**

|  | **This study**  **Pan_*cytb*_SYBR(C̅_t_)** | **This study**  **Pan_*cytb*_TaqMan (C̅_t_)** |
| --- | --- | --- |
| **18 *P. falciparum* (*P. f*)** | 17^b^ (19) | 17^b^ (19) |
| **8 *P. vivax*** | 8 (20) | 8 (20) |
| **3 *P. ovale*** | 3 (22) | 3 (22) |
| **2 *P. malariae* (*P. m*)** | 1^c^ (39) | 1^c^ (36) |
| **2 *P. f* + *P. m*** | 2 (22) | 2 (22) |
| **80 Negatives** | 0 | 1 (41) |

^a^ Predefined samples based on a consensus of results by routine microscopy, the PCR reference method described by Singh et al., a genus-specific *cytb* PCR, a species-specific 18S PCR, all conventional, and sequencing [1].

^b^ The missing *P. falciparum* was a recurrent (recrudescent) case of low parasitaemia. Previous results: One single ring stage parasite detected by microscopy, positive by *cytb* conventional PCR, and negative by 18S reference PCR [1]. Present result: Negative by *cytb* conventional PCR.

^c^ Previous results for the missing *P. malariae*: Negative by microscopy, but positive by conventional PCR [1]. Present result: Positive by *cytb* conventional PCR.

**Reference**

1. Haanshuus CG, Mohn SC, Morch K, Langeland N, Blomberg B, Hanevik K. A novel, single-amplification PCR targeting mitochondrial genome highly sensitive and specific in diagnosing malaria among returned travellers in Bergen, Norway. Malaria journal. 2013;12:26. doi: 10.1186/1475-2875-12-26. PubMed PMID: 23336125; PubMed Central PMCID: PMC3556099.
