# Supplementary material for: Assessment of malaria real-time PCR methods and application with focus on low-level parasitaemia
Source: PLoS One. 2019 Jul 5;14(7):e0218982. doi: 10.1371/journal.pone.0218982 (PMC6611585; doi:10.1371/journal.pone.0218982)
Supplement: S2 Table — (DOCX) [file pone.0218982.s004.docx]

**S2 Table Amplification efficiencies for real-time PCR methods applying a 10-fold dilution series of reference material**

|  | **Platform^a^** | **Product size** | **Slope^b^** | **R^2^** | **E%^c^** | **Reported E%** |
| --- | --- | --- | --- | --- | --- | --- |
| **This study**_*cytb* | SYBR | 220 bp | -3.415 | 0.995 | 96% | - |
| **This study**_*cytb* | TaqMan | 220 bp | -3.183 | 0.992 | 106% | - |
| **Lefterova**_18S | SYBR | 317 bp | -3.307 | 0.987 | 101% | Not reported |
| **Xu**_*cytb* | SYBR | 430 bp | -4.187 | 0.995 | 73% | 98% |
| **Farrugia**_*cytb* | TaqMan | 203 bp | -3.084 | 0.983 | 111% | 95% |
| **Hofmann**_TARE-2 | SYBR | 93 bp | -4.078 | 0.997 | 76% | 85% |
| **Hofmann**_*var*ATS | TaqMan | 65 bp | -3.613 | 0.999 | 89% | 87% |

^a^ Unspecific SYBR Green dye/Specific TaqMan probe with 6-carboxyfluorescein (6-**FAM**) fluorescent dye.

^b^ The slope values were based on best adjusted standard curves applying four dilutions in the range 2000-2 µl, each dilution run in triplicates. A broader range in the dilution series would have provided more accurate E-values.

^c^ Amplification efficiency (E) % = (10^-(1/-Slope)^ – 1) x 100%.
