# Supplementary material for: Assessment of malaria real-time PCR methods and application with focus on low-level parasitaemia
Source: PLoS One. 2019 Jul 5;14(7):e0218982. doi: 10.1371/journal.pone.0218982 (PMC6611585; doi:10.1371/journal.pone.0218982)
Supplement: S3 Table — (DOCX) [file pone.0218982.s005.docx]

**S3 Table Multivariate logistic regression analysis of factors associated with low parasitaemia (≤1000 copies/rxn)**

|  | **OR crude**  **(95% CI)** | **p-value** | **OR adjusted**  **(95% CI)** | **p-value** |
| --- | --- | --- | --- | --- |
| *Age ≤ 12 months* | 5.73 (1.82-18.04) | 0.001† | 4.86 (0.90-26.36) | 0.042† |
| *Male* | 1.39 (0.55-3.49) | 0.483 | * | * |
| *Not travel outside Dar last 4 weeks* | 2.41 (0.87-6.69) | 0.088 | 2.98 (0.71-12.59) | 0.006† |
| *Not referred from another hospital* | 0.53 (0.18-1.57) | 0.244 | * | * |
| *No antibiotics the last 4 weeks* | 0.18 (0.06-0.58) | 0.003† | 0.91 (0.15-5.62) | 0.282 |
| *No antimalarials the last 4 weeks* | 1.65 (0.62-4.43) | 0.317 | * | * |
| *Length of sickness ≤ 5 days* | 0.16 (0.05-0.47) | 0.001† | 0.11 (0.02-0.84) | 0.006† |
| *No antibiotic treatment in hospital* | – | – | – | – |
| *No antimalarial treatment in hospital* | – | – | – | – |
| *Not given the diagnosis malaria* | 3.96 (1.49-10.53) | 0.004† | 4.92 (1.06-22.80) | 0.032† |
| *Not given the diagnosis septicaemia* | 0.33 (0.10-1.05) | 0.053 | 0.48 (0.08-2.80) | 0.409 |
| *Length of admission ≤ 5 days* | 1.57 (0.62-4.00) | 0.343 | * | * |
| *Died in hospital* | 3.49 (1.00-12.24) | 0.040† | 0.78 (0.05-13.36) | 0.867 |

Abbreviation: OR, odds ratio; 95%CI, 95% confidence interval; Dar, Dar es Salaam.

– Variables not included in the regression analysis due to the extensive numbers of missing values.

* Variables with p-value > 0.1 were not included in the multivariate regression model.

† Significant results (p-value < 0.05)
